# Laparoscopic Resection of a Splenic Artery Aneurism with Spleen Preservation

**DOI:** 10.1155/2020/2873560

**Published:** 2020-01-20

**Authors:** Tiago Correia de Sá, Carlos Soares, Jacinta Queirós, Teresa Mónica Rocha, Manuel Oliveira

**Affiliations:** General Surgery Department, Centro Hospitalar Tâmega e Sousa, Avenida do Hospital Padre Américo, No. 210, 4560-136 Guilhufe Penafiel, Portugal

## Abstract

*Introduction*. Splenic artery aneurisms (SAA) are the third most common aneurysms, with reported incidences up to 10.4%. There is a higher prevalence in women, and most are incidental findings on imaging studies. Symptomatic or SAA larger than 20 mm and aneurysms in pregnant or in women of childbearing age are indications for surgery, because of the increased risk of rupture. Treatment options include endovascular, laparoscopic, and open surgical approaches. *Presentation of Case*. A 50-year-old female patient with nonspecific abdominal pain performed a computed tomography scan and angiography, which revealed a 24 × 20 × 19 mm SAA. After a multidisciplinary discussion, selective laparoscopic excision of the aneurysm was performed, with spleen preservation. Perioperative course was uneventful, and the patient remained asymptomatic. *Discussion*. A multidisciplinary discussion is of major importance in guaranteeing the optimal treatment for any given visceral aneurysm. Not all SAA are amenable to endovascular treatment, and laparoscopic surgery has mostly replaced open procedures. Laparoscopic ligation and resection of the SAA with splenic preservation offers permanent treatment, with a low morbidity and short hospital stay.

**Conclusion:**

Selective laparoscopic aneurysm resection is a safe and effective approach, with good short- and long-term results, allowing permanent treatment of SAA while maintaining splenic function.

## 1. Introduction

The splenic artery is the most common visceral artery affected by aneurysms and pseudoaneurysms, and only less frequent than aortic and iliac aneurysms, with reported incidences between 0.02% and 10.4% in the general population. Most splenic artery aneurysms (SAA) are asymptomatic and incidental findings on imaging studies [1]. Prevalence is higher in women and in cirrhotic patients may rise to 8.8% to 50% [1]. The aetiology remains unclear, but several conditions have been associated with SAA, including atherosclerosis, pancreatitis, splenomegaly, portal hypertension, abdominal trauma, pregnancy, and inflammatory and infectious diseases [1].

SAA are typically saccular and mostly located in the distal third of the artery, followed by the medial third, and only rarely in the proximal third or intrasplenic [2]. They contain a variable amount of mural thrombus and are frequently calcified, which is not a protective factor against rupture, as most ruptured SAA are calcified [1].

CT angiography is highly accurate for the diagnosis and characterization of SAA, and 3D reconstructions are usually required to differentiate the false positive of normal vessel tortuosity and atherosclerotic changes.

Indications for SAA treatment remain controversial. Symptomatic SAA, SAA discovered during pregnancy, which have an increased risk of rupture, or in patients of childbearing age, and SAA in liver transplant recipients are indications for treatment, regardless of the size. Most agree that aneurysms larger than 20 mm and enlarging are at an increased risk of rupture and should be treated in all patients with reasonable operative risk and with a life expectancy of 2 years [1, 3, 4].

Treatment options depend primarily on aneurism characteristics, surgical experience and patients' age, operative risk, and comorbidities. Endovascular treatment is currently the preferred approach to SAA with favourable anatomy [3]. Open surgical procedures include ligation, resection, and splenectomy. Several laparoscopic techniques have been described and provide a viable and efficient alternative to the traditional open surgical approach but may be difficult in obese patients, in patients with previous abdominal surgeries, or when the SAA is embedded in the pancreatic parenchyma or deep in the splenic hilum [1].

We report a case of a successful selective laparoscopic resection of a SAA. This case report has been reported in accordance with the SCARE criteria.

## 2. Case Report

A 50-year-old Caucasian female patient was referred by the family physician to the Hepatobiliopancreatic and Splenic (HBPS) Surgery Consult due to a 20 mm SAA. The patient had been complaining of a nonspecific pain in the upper quadrants of the abdomen for months. No other complaints were recorded. Past medical history revealed arterial hypertension and dyslipidaemia, medicated with an angiotensin-converting enzyme inhibitor and with a statin. She had no smoking or relevant alcoholic habits, and she had no past surgical interventions. Family history was irrelevant. No abnormalities in the laboratory studies were identified. She had been previously submitted to an upper endoscopy, which revealed no abnormalities.

**Figure 1:**
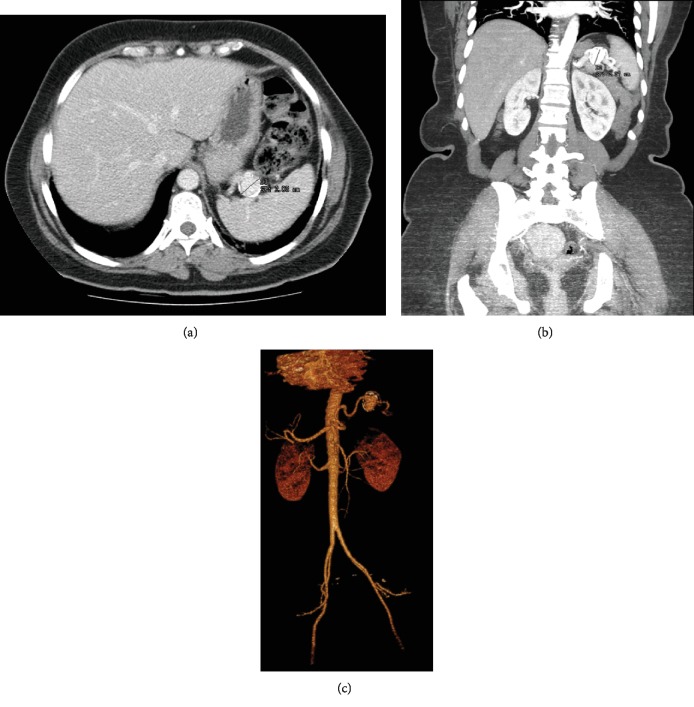
A 50-year-old female patient with an angioCT scan showing a splenic artery aneurism. (a) Transversal cut section. (b) Coronal cut section. (c) 3D angioCT reconstruction.

After careful patient examination and exam reviewing, we conducted an angioCT scan which revealed a SAA with 24 × 20 × 19mm in size, with two branches of the splenic artery originating in the aneurysmal sac (Figure 1). No other abnormal findings were evident. After conjoint evaluation by the Surgical, Interventional Radiology, and Vascular Surgery Departments, a laparoscopic aneurysmectomy was preferred, due to the unfavourable anatomy of the SAA for an endovascular approach. Three weeks prior to the procedure, the patient was vaccinated against Streptococcus pneumoniae, Haemophilus influenzae type B, and Neisseria meningitides, to reduce the risk of overwhelming postsplenectomy sepsis, if splenectomy was necessary.

The procedure was performed under general anaesthesia. The patient was placed in the supine position. The positioning of the trocars was as follows: 12 mm trocar at the umbilicus for the camera, a 5 mm trocar in the epigastric area, a 5 mm trocar in the right upper abdomen, and a 12 mm trocar in the left lower abdomen. A pneumoperitoneum of 12 mmHg was created and maintained.

**Figure 2:**
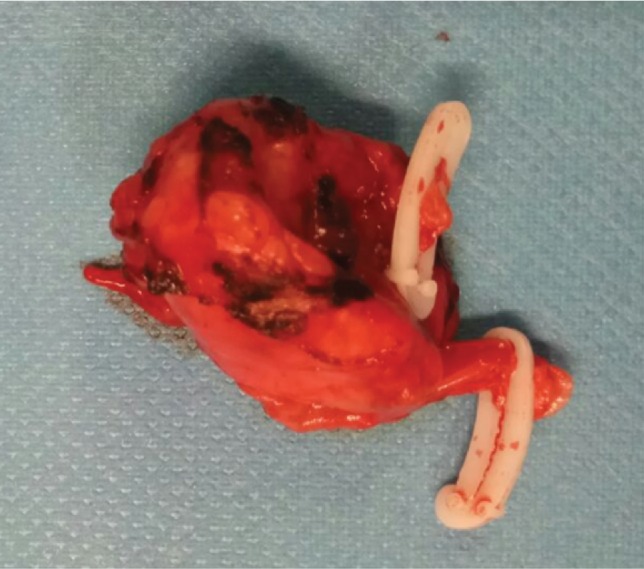
Surgical specimen. Two hemolocks on the efferent branches originating in the aneurysmal sac are seen. Anatomopathological study revealed a saccular aneurysm with 28 × 22 × 19 mm, with mural thrombus, confirming the diagnosis.

A monopolar laparoscopic hook and ultrasonic energy were used for dissection. First, the gastrocolic ligament was divided, and the stomach lifted to expose the anterior pancreatic surface. Special care was taken not to interrupt the short gastric vessels while opening the lesser sac, as this collateral circulation is of major importance in maintaining spleen perfusion. The superior border of the pancreas was approached, and the splenic artery was identified near its origin. This vessel was dissected and looped with an umbilical tape to gain vascular control of the splenic artery proximal to the aneurysm. After dissection of the splenic artery, the aneurysmal sac was isolated. One afferent branch and two efferent branches were identified and ligated with hemolocks. Aneurysmectomy was performed. A few minutes after, the superior pole of the spleen appeared dusky but returned to normal colour at the end of the procedure. It was decided not to pursue with splenectomy. Haemostasis was evaluated, and a multitubular drain was placed. The wounds of the abdominal incisions were closed. Operating time was approximately 120 minutes, and the estimated blood loss was 120 ml. Postoperative course was uneventful, with drain removal on the second postoperative day. The patient was discharged home on the 4^th^ postoperative day. Postoperative amylase levels were within the normal range, and there were no clinical signs of pancreatitis nor pancreatic leak. Anatomopathological study of the resected aneurysm confirmed the diagnosis, revealing a saccular aneurysm with 28 × 22 × 19 mm, with mural thrombus (Figure 2).

**Figure 3:**
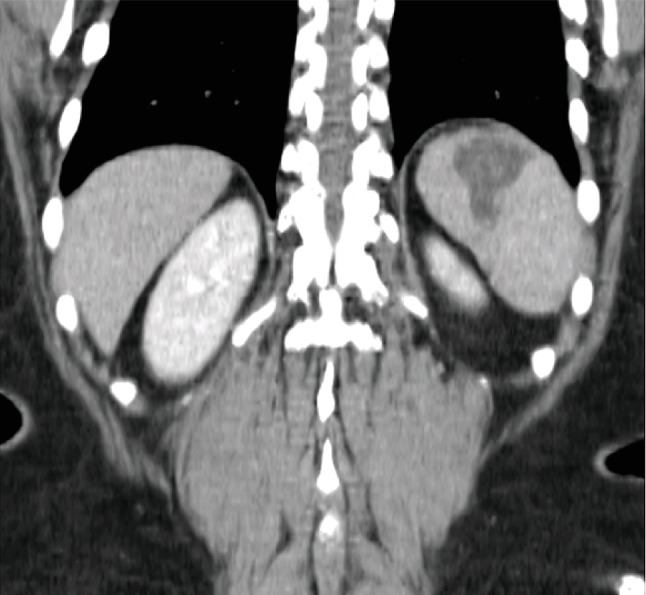
First postoperative CT scan showing an ischemic area of 40 × 40 mm in the superior pole of the spleen.

The patient was asymptomatic at the first month follow-up consultation, and laboratory studies revealed no abnormalities. The postoperative abdominal CT scan revealed a 40 × 40 mm area of infarction in the superior pole of the spleen, representing less than a third of the total parenchyma volume (Figure 3). At the 3-, 6-, and 12-month follow-up reevaluation, the patient remained asymptomatic with no laboratory abnormalities and similar imaging findings. As so, no further interventions were proposed, and the patient was discharged from the HBPS consult and is now regularly evaluated by her family doctor.

## 3. Discussion

There are several surgical approaches described for SAA, but few published cases of SAA ligation and resection with spleen preservation.

Due to technical advances and liberal use of CT imaging, SAA incidence is increasing [3]. This patient was referred to our unit after a CT scan, performed for the study of an unspecific abdominal pain, revealing a SAA.

Despite its low incidence and mostly asymptomatic course, SAA rupture can be fatal [5]. There is no clearly defined size cut-off for treatment, although 2 cm is commonly cited [1, 3, 6]. However, symptomatic SAA and SAA discovered during pregnancy, in patients of childbearing age, and in liver transplant recipients are indications for surgery [1, 3, 4]. Our patient had a possibly symptomatic SAA, 24 × 20 × 19 mm in size, and surgical treatment was proposed. To our knowledge, there are no guidelines or consensus for treatment of SAA. On the other hand, there are several effective treatment alternatives. Endovascular approaches, with transcatheter embolization, have been preferred, due to their low morbidity and mortality. However, not all aneurysms are suitable for this technique. A multidisciplinary discussion is of major importance in guaranteeing the optimal treatment for any given visceral aneurysm. After the conjoint evaluation, a laparoscopic approach was decided, as artery tortuosity and the distal localization of the aneurysm made the endovascular approach less feasible.

Several laparoscopic approaches have been described [7]. A tangential stapler resection of sacciform aneurysms to preserve splenic flow was initially described but may lead to recurrence [8]. Proximal and distal ligations, with or without resection of the SAA and with or without splenectomy, are safer approaches [3, 6, 9]. In experienced hands, this is a simple, safe, and minimally invasive technique, allowing rapid recovery, decreased postoperative pain, and shorter hospital stay than open surgery [6, 10]. Reconstruction of vascular continuity of the splenic artery is usually not necessary due to the collateral circulation provided by the short gastric vessels. End-to-end anastomosis have been described, particularly in young patients, where spleen preservation is advisable [6].

We proposed laparoscopic ligation and resection of the SAA, with splenic preservation. To our knowledge, few cases of aneurysmectomy with spleen preservation have been published [11, 12]. Advanced laparoscopy skills are of major importance, as dissection may be difficult. Early in the procedure, proximal isolation of the splenic artery is crucial, allowing subsequent vascular control, should this need arise. Distally located aneurysms are more difficult to dissect, and in this location, the splenic vein may be damaged, as small branches may be in contact with arterial branches. However, most SAA are distal and less frequently favourable to endovascular treatment [10, 12].

The risk of pancreatic injury during laparoscopic dissection may be decreased compared to open surgery, by avoiding retractors and direct manipulation of the pancreas. Also, the pancreas and splenic artery are generally separate, and a plane of dissection can usually be found between the two.

Laparoscopic ultrasonography with Doppler function is not indispensable but may be useful to evaluate splenic arterial flow signs before and after SAA exclusion [6]. In particular, distal SAA are more frequently associated with a drop in splenic arterial flow after exclusion than more proximal aneurysms, where short gastric arteries and the intrapancreatic network can bypass and avoid a drop in arterial flow. The simple darkening of the surface of the spleen does not necessarily imply that the spleen will develop an infarction, and there is evidence that after surgery, the collateral circulation is capable of reestablishing the flow to the spleen improving the splenic perfusion [6]. Areas of splenic infarction inferior to one third of the splenic parenchyma may be well tolerated and require no intervention, as described by Warshaw in distal pancreatectomies with splenic vessel transection [13]. In fact, these patients developed areas of splenic infarction in 22% of cases, but only 2% required surgery [13]. Our patient developed an area of splenic infarction inferior to a third of the total splenic parenchyma volume, and no intervention was required. As the patient remained asymptomatic during 1 year of follow-up, with similar imaging findings, she was discharged from the HBPS Surgery Consult to the family physician.

## 4. Conclusion

SAA have been increasingly diagnosed with the liberal use of imaging studies. Currently, there are no treatment guidelines nor consensus for SAA. A multidisciplinary approach is of major importance in the management of all visceral aneurysms. A predominantly distal location and splenic artery tortuosity make SAA not always amenable to endovascular treatment. Surgery is an important treatment modality, and several laparoscopic approaches have been described. Selective laparoscopic ligation and resection of SAA with spleen preservation is a safe treatment modality for selected cases, with good short- and long-term results, allowing permanent treatment of SAA while maintaining splenic function.
